# Electrical grid linked to PV/wind system based fuzzy controller and PID controller tuned by PSO for improving LVRT

**DOI:** 10.1038/s41598-025-87208-z

**Published:** 2025-03-05

**Authors:** Noura G. EL Sayed, Ali M. Yousef, Gaber El-Saady, Meshari D. Alanazi, Hamdy A. Ziedan, Montaser Abdelsattar

**Affiliations:** 1 Department of Electrical Engineering, Faculty of Engineering, Assiut University, Assiut, 71516 Egypt; 2 Department of Electrical Engineering, College of Engineering, Jouf University, 72388 Sakaka, Saudi Arabia; 3 Department of Electrical Engineering, Faculty of Engineering, South Valley University, Qena, 83523 Egypt

**Keywords:** Power quality, Hybrid system, Wind energy, Photovoltaic (PV), Static synchronous compensators (STATCOM), Low voltage ride through (LVRT), Proportional—integral—derivative (PID) control, Fuzzy logic control (FLC), Praticle swarm optmization (PSO), Engineering, Electrical and electronic engineering

## Abstract

This paper presents the use of a static synchronous compensators (STATCOM) device to improve the low voltage ride through (LVRT) ability of an electrical network consisting of wind farms that produce 9 MW and 1 MW PV stations during grid faults. A hybrid energy model is connected with 100 MVAR STATCOM at the point of common coupling (PCC) through line to line fault occurs on the grid. STATCOM control is used to detect the voltage at the PCC bus through occurring line to line (LL) faults by compensating reactive energy. A method of particle swarm optimization (PSO) is utilized for adjusting the optimum value of proportional—integral—derivative (PID) STATCOM control. STATCOM is controlled by (PID) and is compared with STATCOM controlled by fuzzy logic control (FLC). The proposed system has been performed utilizing Matlab/Simulink. Results of the simulation clear effectiveness and the ability of STATCOM with FLC in improving LVRT, power quality, and mitigation voltage dip, during grid faults like line to line (LL) faults as compared with STATCOM with PID control.

## Introduction

The request for energy production in these days has increased, production of many renewable energy sources and using of nonlinear loads are increasing. Solar energy is counted as one of the most installed renewable sources of power because it is environmentally friendly and its operating costs are small^1^. Renewable sources of power supply an alternative to meet the needs of electrical power and reduce the environmental effect, with the emergence of solar power as a prominent source of renewable power to integrate power^2–4^. Photovoltaic (PV) cells are playing a vital role in transforming solar power into electrical power^5–7^. Renewable power sources rise quickly in wind power^1–8^. Wind energy installed capacity production worldwide reached 743 GW at the end of 2020 that act about 26 percent of renewable power production^9^. Wind energy conversion systems (WECS) are based on wind turbines of variable and fixed speed. The fixed speed of wind turbine formation was modest but it was not able to obtain the maximum energy at changing speeds of wind where its slip could only be changed over a short range^8–10^. Doubly Fed Induction Generator (DFIG) is critical to faults in the grid because the stator of DFIG is directly linked to power systems such as reactive power control, independent active, variable speed capability, lower active power losses, and lower converter cost^11^. When a grid fault occurs, the transient flux of the stator establishes the surge current in the circuit of a rotor that can damage the converter. The dip of voltage occurs at PCC and decreases the converter’s ability to transport energy to an electrical network causing rising DC-link capacitor voltage^12^.

Reference^13^ improved power management control method of a hybrid Direct Current (DC) Micro-Grid (MG) system was proposed. It consists of a PV cell, battery energy storage, wind turbine, fuel cell, and electrolyzer. The control of battery energy storage is the PI controller. The gains of the PI controller are tuned by utilization control of Takagi–Sugeno (TS)-fuzzy to obtain a better dynamic voltage response of the DC MG system. In Ref.^14^ hybrid technique by combining an evolutionary optimization technique was proposed. The modified invasive weed optimization (MIWO) method with conventional perturb and observe algorithm to improve search performance for the maximum power output of PV system. In Ref.^15^ the control method and optimization method were presented. A hybrid Firefly Algorithm-Particle Swarm Optimization (FA-PSO) was utilized. Takagi Sugeno Fuzzy Inference Systems (TSFIS), Adaptive Neuro-Fuzzy Inference Systems (ANFIS), and Fractional Order Proportional Integral-Derivative (FO-PID) control. The results showed the superiority of the (FA-PSO) optimized ANFIS-PID controller method compared to fuzzy PI and PID controller.

In Ref.^16^ the presented method of the strategy of demagnetization control and external resistance in the DFIG rotor and stator side to enhance the LVRT capability of the DFIG-based wind energy conversion system. The external resistance in the stator side accelerated the damping of transient flux by decreasing constant time. For demagnetizing control, transient responses of electromagnetic torque, DC-link voltage, stator, and rotor current, of DFIG were improved at initiation and redemption at grid faults. Ref.^17^ presented designing the construction control of hybrid system PV and wind linked to grid. The maximum Power Point Tracking method was utilized to obtain maximum power from the subsystem. The control method adjusted DC link voltage, maintained the voltage of the grid constant, and controlled power fed into the grid. In Ref.^18^ the deloading based on active power control of variable speed DFIG wind turbines was introduced for adjusting primary frequency. To deal with a nonlinear curve of power characteristics for wind turbine generators, a Lagrange interpolating polynomial was introduced. The suggested method of Lagrange interpolating polynomial based on deloading, the overall capacity factor of the wind farm was enhanced, and the fuel consumption of the diesel generator reduced for the same power demand. Ref.^19^ introduced the controlling abilities of a large PV farm as a solar-PV inverter to mitigate torsional oscillations, electromechanical, and chaotic electrical. A solar-PV inverter operated as a PVSTATCOM to stabilize different modes of a Turbo generator-based on power system. An intelligent maximum power point tracking control of the DC-Link capacitor voltage was proposed. Ref.^20^ presented a comprehensive performance assessment of the two metaheuristic Bacterial Foraging Optimization Algorithms (BFOA), Particle Swarm optimization (PSO) algorithms, and hybrid PSO-BFOA optimizer for controlling the oscillations of power in a two-area four generator system integrated with a large-scale PV-farm. PV-plant operated as Voltage Source Converter-STATCOM to improve stability of the power system. In Ref.^21^ an intelligent Wind–PV-STATCOM was proposed for overall stability improvement, including the conventional zero mode stability and the torsional mode simultaneously. The gate signal in Wind–PV-STATCOM was controlled with an innovative controller that regulates the DC and AC-currents. Ref.^22^ proposed a method of controlling the power flow of PV-STATCOM by optimization methods like Bacterial Swarm Optimization (BSO), a hybrid of particle swarm and bacterial foraging optimizations.

STATCOM was used to compensate reactive power demand of the load, so it improves voltage regulation of the source^23^. STATCOM was controlled for absorbing or injecting reactive power to control network voltage, also it solved the problem of stability and harmonic^24^. STATCOM was controlled by a fuzzy logic-based PI control and a result has been compared with classic control under grid faults. The method of PSO was used to detect the parameter of PI control. The stability of voltage was defined as the capacity of the energy system to preserve the stability of voltage at buses in the system after a disturbance of the grid occurred. On the contrary, the instability of voltage expresses the defeat of the energy system to preserve voltage steady at its buses after grid troubles occur^11^.

In Ref.^25^ The LVRT problem occurred when it is near the fault of the grid. In this case, it causes a reducing in the voltage of the grid when the point of the generator is connected to the grid this leads to limited power that can be generated from the device.

LVRT was used when a voltage-sagging state existed. It can preserve control over the system under such a process^26^. The STATCOM can adjust the voltage of the grid at (PCC) by compensating a specific quantity of reactive energy into the voltage of source converters by utilizing power storage. It detects the magnitude of voltage and modulates phase angle in a very short term that enhances signal quality^27–30^. STATCOM is used to enhance the LVRT capacity of DFIG based on wind in dynamic conditions. Method of optimization such as PSO, Water Cycle Algorithm (WCA), and hybrid optimization of PSO and WCA were utilized to improve the performance of STATCOM^28^. Faults on DFIG are very dangerous. So crowbar and chopper circuits are used to protect DFIG from grid faults. However, during crowbar operation and absorbing reactive power from the grid, DFIG becomes an asynchronous motor, which leads to damage to the voltage of the grid. So STATCOM was utilized to compensate reactive power to improve terminal voltage through faults and enhancement power quality^31^. Reference^32^ proposed STATCOM to compensate voltage of the Egyptian energy network linked with the Al Zafarana-5^th^ stage. PI STATCOM controller was compared with the fuzzy STATCOM controller and results cleared that fuzzy STATCOM controller improved swelling of voltage and sagging compared with PI STATCOM control^33,34^. In Ref.^35^ allocation of the hybrid model which contains DSTATCOM and PV-DG was proposed, also the MPA optimization method was introduced. Results of hybrid DSTATCOM and PV-DG models cleared improving profile of voltage, stability of voltage, and improving economic sketch. Reference^36^ introduced the distribution Static Synchronous Compensator (DSTATCOM), sizing PV, and optimal sitting in the distribution grid East Delta Network (EDN). Ant Lion Optimizer (ALO) was introduced to improve the quality of power of the network. DSTATCOM was used for minimizing power losses and improving the profile of voltage, and stability of the system. Reference^37^ proposed modeling and design that used a series resistor protection method for improving LVRT. The protection method was designed with an assortment of variables of a system containing DC link voltage, fluctuations, the current of the rotor, and reactive and active energy. Reference^38^ presented that STATCOM was used to compensate for reactive power, improve LVRT, and improve the dynamic performance of the network linked to the wind energy plant. STATCOM was tuned by utilizing the WCA, PSO, and (WCA-PSO) hybrid algorithm. Reference^39^ introduced a grasshopper optimization algorithm (GOA), PSO, and (GOA-PSO) hybrid algorithm for improving LVRT, minimizing loss of generation through faults, and reducing oscillations. In Ref.^40^ a cost-effective control method was presented to improve LVRT and smooth output power of a three-phase multi-level Flying Capacitor Inverter (FCI) in a wind turbine based on a permanent magnet synchronous generator (PMSG). The presented method of flying capacitors reduces fluctuation and sagging of voltage. Reference^41^ proposed a coordinated control strategy for a Voltage source converter (VSC)-based on multi-terminal direct current (MTDC) for improving LVRT and frequency. In Ref.^42^ demagnetization control method was proposed for eliminating the problem of magnetism and improving LVRT.

In Ref.^43^ STATCOM was proposed to improve hybrid system performance that includes wind system and PV systems through three-phase faults happening at PCC among hybrid system and networks. STATCOM was based on two PI control to regulate reactive power among hybrid system and STATCOM at PCC and improve voltage. A comparison between the whale optimization algorithm (WOA) and (PSO) was introduced where WOA gave better performance than PSO. In Ref.^44^ STATCOM was presented for improving the profile of voltage, sagging of voltage, improving transient voltage stability, and helping wind turbine systems maintain service through faults of the grid. This paper studies STATCOM devices to improve power quality, LVRT problems, and dynamic performance for electrical grids connected to hybrid systems containing PV and wind farms. STATCOM is controlled by PID controller and is compared with FLC STATCOM. The PSO optimization method is utilized to detect the parameters of the PID controller. The results proved the effectiveness of STATCOM in compensating voltage dip, active power, and protect DC-Link voltage from oscillation. This paper presents a motivation for enhancing the LVRT problem and its impact on in electrical grid linked to PV/wind hybrid systems during grid faults. STATCOM device is applied on an electrical grid connected with the hybrid system at the PCC bus to adjust the voltage by compensating reactive power. PID STATCOM and FLC STATCOM are applied to improve LVRT and power quality. Also, the method of PSO optimization is utilized to detect the parameter of the PID controller to decrease the error signal. LVRT issue happens due to asymmetric faults like line to line fault that stratifies in this system. Finally, the main contributions of this paper are briefed as follows:

- Discussing the issue of LVRT, and its impact on the electrical grid linked to a hybrid system containing 1 MW PV station and 9 MW wind farm.
- Introducing the STATCOM controller to improve LVRT capability and power quality
- Showing STATCOM that is based on a PID controller and fuzzy logic controller for improving voltage, compensating reactive power, and protecting the voltage of the DC link from oscillation
- Showing PID control that is tuned by the PSO method to adjust the parameter of PID control and reduce the error signal.
- Presenting an inclusive comparison between FLC STATCOM and PID STATCOM to improve LVRT capability.
- Introducing the Simulation results to clear the effectiveness of STATCOM in mitigation voltage dip, compensating active power of PV stations and wind farms, and protecting DC-Link voltage from overvoltage.

This paper is organized as follows; “System construction” section presents System construction. “Fuzzy logic control” section introduces Fuzzy Logic Control. “PID controller” section provides a PID controller. “Particle SWARM optimization” section introduces Swarm optimization, “PID controller based PSO” section introduces STATCOM configuration and control circuit of STATCOM, “STATCOM configuration” section introduces simulation study and results, and “Simulation study and results” section presents the conclusion.

## System construction

**Fig. 1:**
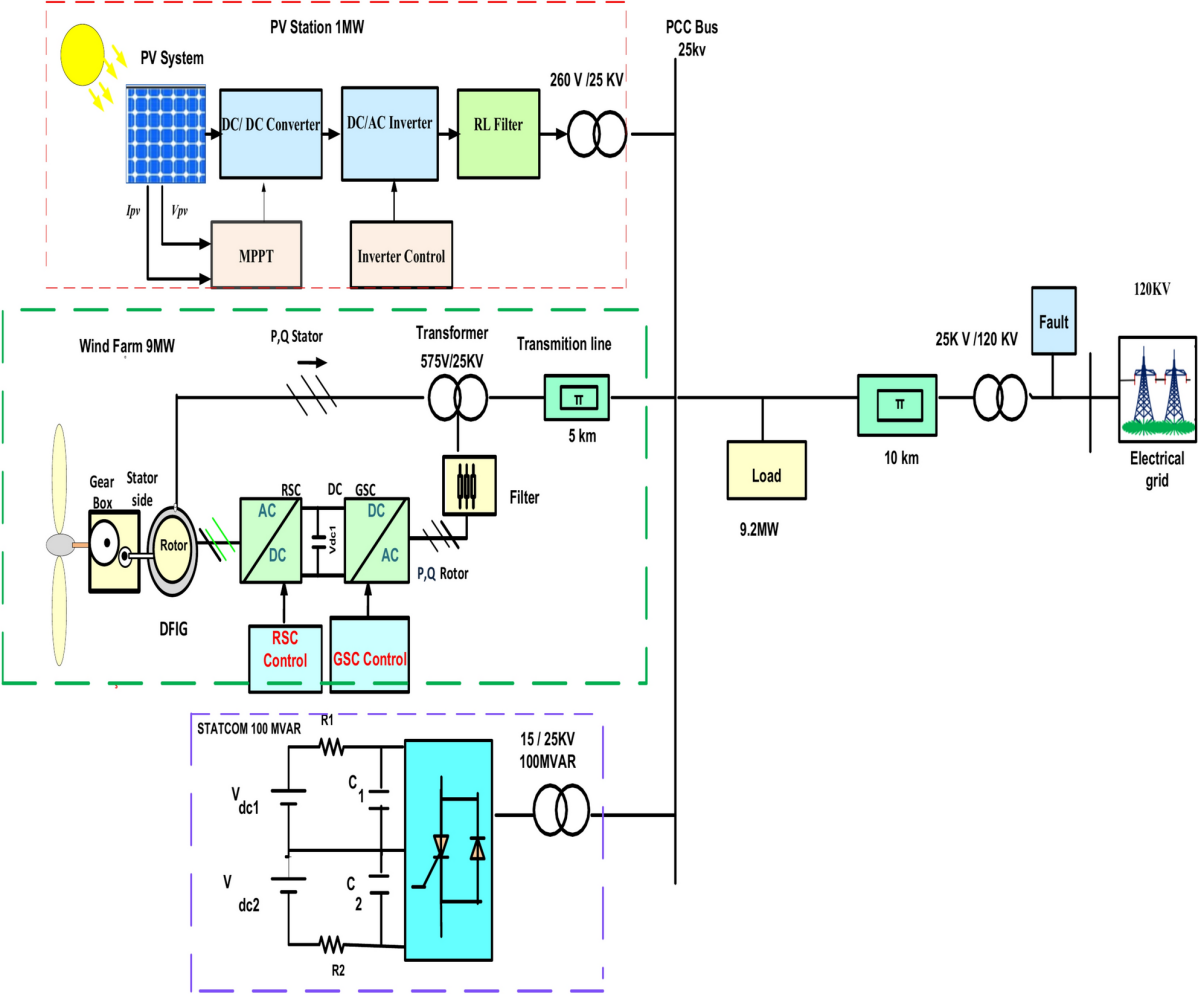
PV/wind hybrid renewable power system configuration with STATCOM.

The presented hybrid energy system consists of a 1 MW PV farm and 9 MW DFIG wind turbine linked to the network as cleared in Fig. 1 and STATCOM is linked to network at PCC bus, the wind farm generates 9 MW^45^. Also, a PV station that generates 1 MW contains 660 parallel-linked PV strings; each PV string consists of five PV panels linked in series. STATCOM is utilized in the system for enhancing LVRT ability and improving the stability of voltage during occurring faults on the electrical grid^46^. The parameters of model is shown in Table 1.

**Table 1:** Suggested hybrid system and STATCOM parameters.

Wind farm parameters	
Rated power of wind farm	9 MW
Rated power of wind turbine	1.5 MW
Number of turbine	Six
Generator type of turbine	DFIG
DC bus voltage	1150 V
Stator resistance	0.023
Stator leakage inductance (pu)	0. 18
Rotor resistance	0.016
rotor leakage inductance (pu)	0.16
Magnatizing inductance (pu)	2.9
Rated stator line-to-line voltage (V)	575
Cut-in wind speed	4
Rated wind speed	12
Cut-out speed (m/s)	20
PV station parameters	
Rated power of PV station	1 MW
Rated power of PV panel	315.072 W
Open circuit voltage	64.6 V
Short circuit current of PV panel	6.14 A
Rated power of PV panel	315.072 W
Standard test condition	G = 1000 W/m^2^ ,T = 25, 45 °C
DC link voltage	500 V
Number of parallel-connected PV strings	640
Number of series-connected PV panels per string	5
Nominal primary and secondary voltages (V)	[25e3 260]
STATCOM parameters	
Secondary nom. voltage phase shift	[15e3 + 7.5]
Resistance of zig-zag winding [R L] (pu)	[0.05/30]
Primary (zig-zag) nominal voltage Vp (VrmsPh-Ph)	25e3/4
Capacitance	3000 μF
Number of zigzag phase-shifting transformer	4
Rated power of STATCOM	100 MVAR
Magnetizing resistance (pu)	500
Magnetizing inductance (pu)	500
Grid parameters	
Rated voltage	120 kV
Load parameter	
Active power of load	9.1 MW
Reactive power of load	2 MW

### PV station model

**Fig. 2:**
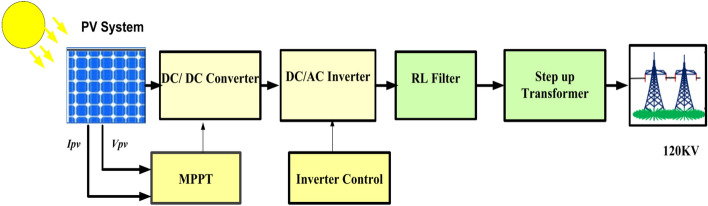
Block diagram of PV model linked to grid.

**Fig. 3:**
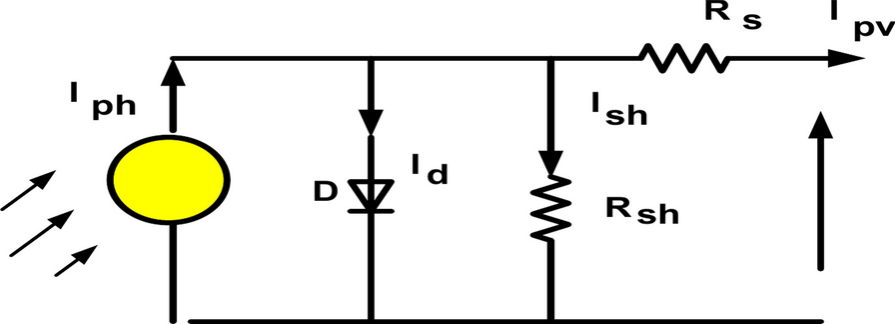
Equivalent circuit of single diode model.

Among the abundant renewable energy resources considered PV is the best alternative choice for producing electrical power from solar energy without emission of greenhouse gases, low maintenance, long lifespan, and good efficiency^47^. The simplified circuit model of the PV cell is shown in Fig. 2. It contains a series resistor, photodiode, and current of the source. The dynamic attitude of the PV model is acted by eight differential equations, which characterize the cell of PV, DC/DC converter, Low Pass (LP) filter, and the Voltage Source Inverter (VSI)^48,49^. Figure 2 shows a block diagram of the PV model linked to grid and Fig. 3 shows an equivalent circuit of a single diode model of the PV system^50^.

1 $$I_{pv} = I_{ph} - I_{d} - I_{sh} = I_{ph} - I_{o} \left[ {\exp \left( {\frac{{V_{ph} + I_{pv } R_{s } }}{{\text{a}}}} \right) - 1} \right] - { }\frac{{V_{ph} + I_{pv } R_{s } }}{{R_{sh} }}$$

2 $${\text{a}} = \frac{{{\text{nkT}}}}{{\text{q}}}$$

where ${I}_{pv}$, ${V}_{ph}$: Current, and voltage of PV panel; ${R}_{s }, {R}_{sh}$: Series, shunt resistances respectively; $a$, K: Ideality factor, Boltzmann constant (1.38 × 10^−23^ J); n, T: Number of cell in series, Temperature of PV cell; q: Electron charge (1.602 × 10^−19^ C); ${I}_{ph}$, ${I}_{o},{I}_{d}$: Photocurrent and reverse saturation current, and diode current.

### DFIG model

**Fig. 4:**
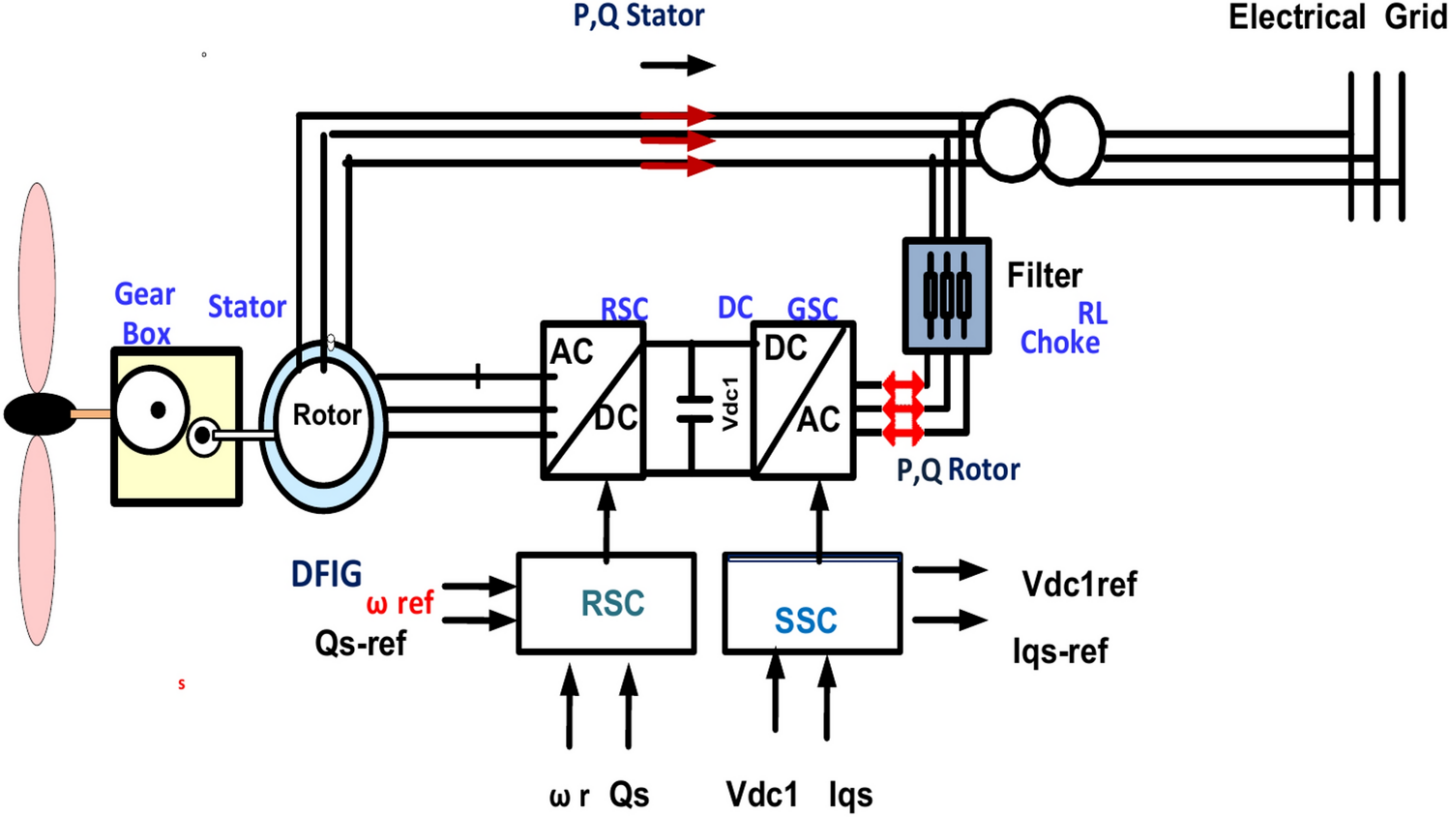
Configuration of wind system.

DFIG has advantages such as controlling active and reactive power, low converter rating, variable speed capability, and low initial cost. However, DFIG is sensitive to grid faults^51^. The stator of DFIG linked immediately to the network, however, the rotor of DFIG was linked to the network during the power converter, as cleared in Fig. 4^52^ The Grid Side Converter (GSC) preserves the voltage of the DC link constant while the Rotor Side Converter (RSC) controls reactive and active power flow from the stator to the network^53^. The following equation describes the output of mechanical power^54^. The wind system is connected with the PCC bus during 0.575/25 kV Δ/Y step-up transformer to inject produced energy into the electrical network. Figure 4 shows the configuration of the wind system.

3 $$P_{m} = \frac{1}{2}C_{p} \left( {\lambda ,\beta } \right)v_{w}^{3} \rho A_{t}$$

Relation among tip speed ratio $\lambda$ and pitch angle of blades (β) can describe by the following equations:

4 $$C_{p} \left( {\lambda ,\beta } \right) = {\text{C}}1\left( {\frac{{{\text{C}}2}}{{\lambda_{i} }} - {\text{C}}3\beta - {\text{C}}4} \right){\text{exp}}^{{\left( {\frac{{ - {\text{C}}5}}{{\lambda_{i} }}} \right)}} + C6\lambda$$

5 $$\frac{1}{{ \lambda_{i} }} = \left( {\frac{1}{ \lambda + 0.08\beta }} \right) - \left( {\frac{0.035}{{ \beta^{3} + 1}}} \right)$$

6 $$P_{m} - {\text{Pe}} = \frac{{2H_{g} }}{{\omega_{s} }}\frac{{d_{\delta }^{2} }}{{d_{t}^{2} }}$$

where ${P}_{m},\text{Pe}$: Mechanical and electrical power of wind; ${C}_{p}$, ${v}_{w}$, $\rho$, ${A}_{t}$: Wind performance coefficient, velocity of wind, density of air, and area swept out by blades of wind turbine; $\lambda ,\beta {,H}_{g}$: Tip speed ratio of rotor blade and blade pitch angle, Inertia constant.

## Fuzzy logic control

**Fig. 5:**
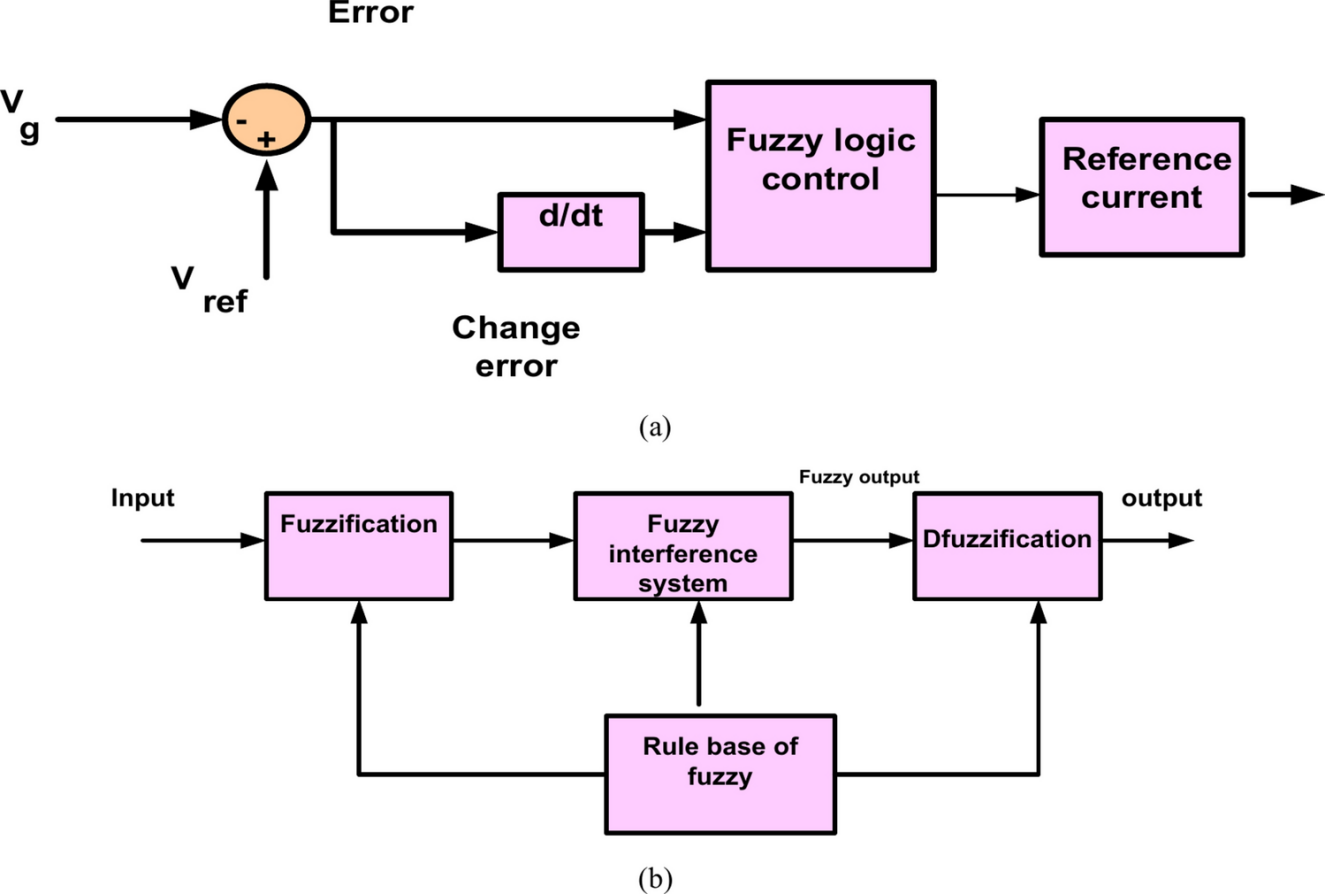
(**a**) Fuzzy logic system, (**b**) flow of FLC system.

**Fig. 6:**
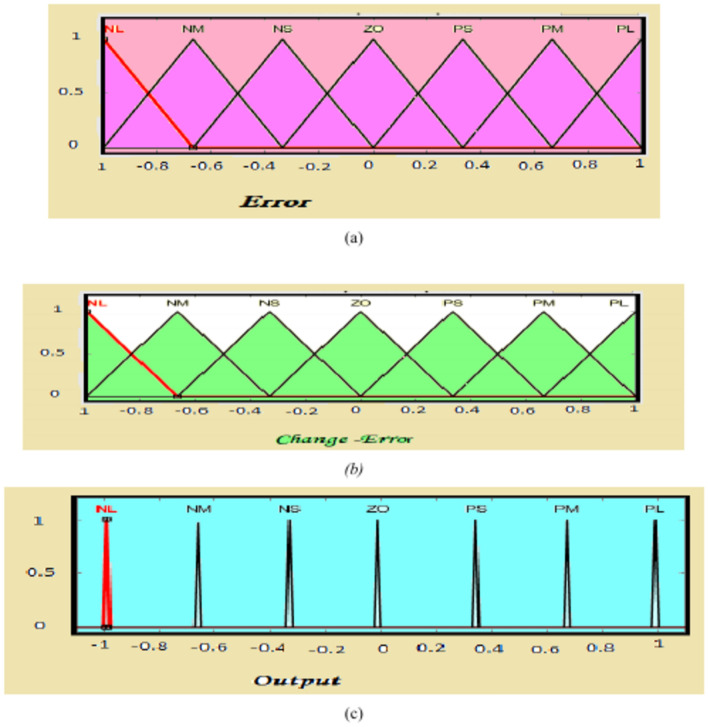
(**a**) Variable input error. (**b**) Variable input change-error. (**c**) Variable output*.*

**Fig. 7:**
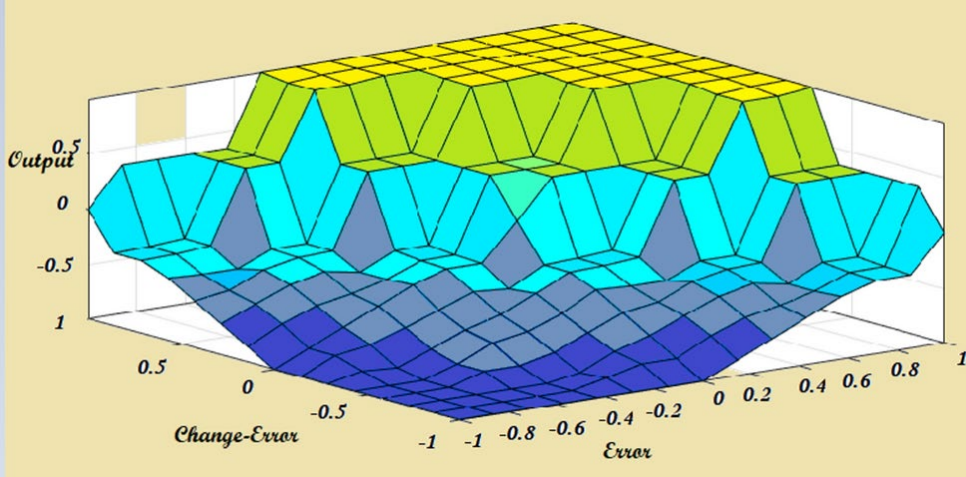
Surface view of FLC.

Figure 5 shows a Fuzzy Logic System (FLC). FLC is a proficient system, it tries to perform human knowledge in the shape of rules. FLC is composed of four parts, called, Fuzzification, knowledge base, inference, and defuzzification. FLC has two input errors and a change error as well as output. The input and output variables were fuzzified by utilizing seven fuzzy sets Negative Large (NL), Negative Medium (NM), Zero (ZO), Negative Small (NS), Positive Small (PS), Positive Medium (PM), and Positive Large (PL). The normalized membership functions for input and output variables as cleared in Fig. 6, and the surface view of FLC is cleared in Fig. 7. Table 2 shows knowledge that is represented in the shape of rules^55,56^.

**Table 2:** Rule base for FLC.

Error	Change error						
	NL	NM	NS	ZO	PS	PM	PL
PL	ZO	PS	PM	PL	PL	PL	PL
PM	NS	ZO	PS	PM	PL	PL	PL
PS	NM	NS	ZO	PS	PM	PL	PL
ZO	NL	NM	NS	ZO	PS	PM	PL
NS	NL	NL	NM	NS	ZO	PS	PM
NM	NL	NL	NL	NM	NS	ZO	PS
NL	NL	NL	NL	NL	NM	NS	ZO

## PID controller

PID is the simplest controller in construction and linear controller. So it’s considered a classic controller device. PID controller is an important control in many industrial engineering such as electrical and mechanical systems^57^. PID control has three parameters are, for proportional, integral, and derivative control. Duty control to regulate gain coefficients to reduce dynamic responses and error together^58^. The coefficients of the PID controller are shown in Eq. (7).

7 $${\text{u}}\left( {\text{t}} \right) = K_{P} e\left( t \right) + ~K_{i} \mathop {\oint }\limits_{0}^{t} e\left( {\mathop {{\text{ }}t}\limits^{\prime } } \right)d\mathop {{\text{ }}t}\limits^{\prime } + ~K_{D} \mathop {\oint }\limits_{0}^{t} e(t)/dt$$

## Particle SWARM optimization

Ebrhart and Kenedy 1995 designed the method of Particle Swarm Optimization (PSO) based flocking of birds’ social attitudes^37,38^. The principle operation of PSO is described as follows: Firstly the location of the domestic particle in the search region is updated according to the next equation^50,59^.

8 $${X}_{k+1}^{\text{i}}={X}_{k}^{\text{i}}+{V}_{k+1}^{\text{i}}$$

where calculation of particles velocity ${V}_{k+1}^{\text{i}}$ was calculated by

9 $${V}_{k+1}^{\text{i}}={V}_{k}^{\text{i}}+{C}_{1}{{r}_{1}(P}_{k}^{\text{i}}-{X}_{k}^{\text{i}})+{C}_{2}{{r}_{2}(P}_{k}^{\text{g}}-{X}_{k}^{\text{i}})$$

where ${V}_{k}^{\text{i}}$ is particle velocity, ${X}_{k}^{\text{i}}$ is location of particle, ${P}_{k}^{\text{g}}$ is swarm optimal location, ${P}_{k}^{\text{i}}$ is optimal position of specific particle, ${C}_{1}$ and ${C}_{2}$ are parameters of social and cognitive, also ${r}_{1}$ and ${r}_{2}$ are selected random numbers between zero and one.

Succession of investigation PSO method can be proved by the next stages.

i. **Initialization**
1) Adjust the particles (K) constants number ${C}_{1}$ and ${C}_{2}$.
  2) Initialize the positions of particle at random.
  3) Initialize the velocities of particle at random.
  4) Set iter = 1.
ii. **Optimization**
  1. Estimate the value of function ${F}_{k}^{\text{i}}$ using designing space of coordinates ${X}_{k}^{\text{i}}$
  2. If $${F}_{k}^{\text{i}}$$ ≤ $${F}_{best}^{\text{i}}$$then $${F}_{best}^{\text{i}}$$ = $${F}_{k}^{\text{i}}$$ , $${P}_{k}^{\text{i}}$$ = $${X}_{k}^{\text{i}}$$ .
  3. If $${F}_{k}^{\text{i}}$$ ≤ $${ F }_{g}^{\text{i}}$$then $${ F }_{best}^{\text{g}}$$ = $${F}_{k}^{\text{i}}$$ , $${P}_{k}^{\text{g}}$$ = $${X}_{k}^{\text{i}}$$ .
  4. If stopping status is convinced, then go to step 3.
  5. Update all the particle velocities if ${V}_{k}^{\text{i}}$ for i = 1..... P
  6. Update all the positions of particle if ${X}_{k}^{\text{i}}$ for i = 1..... P.
  7. Increment K
  8. Go to (1).
  9. End

**Fig. 8:**
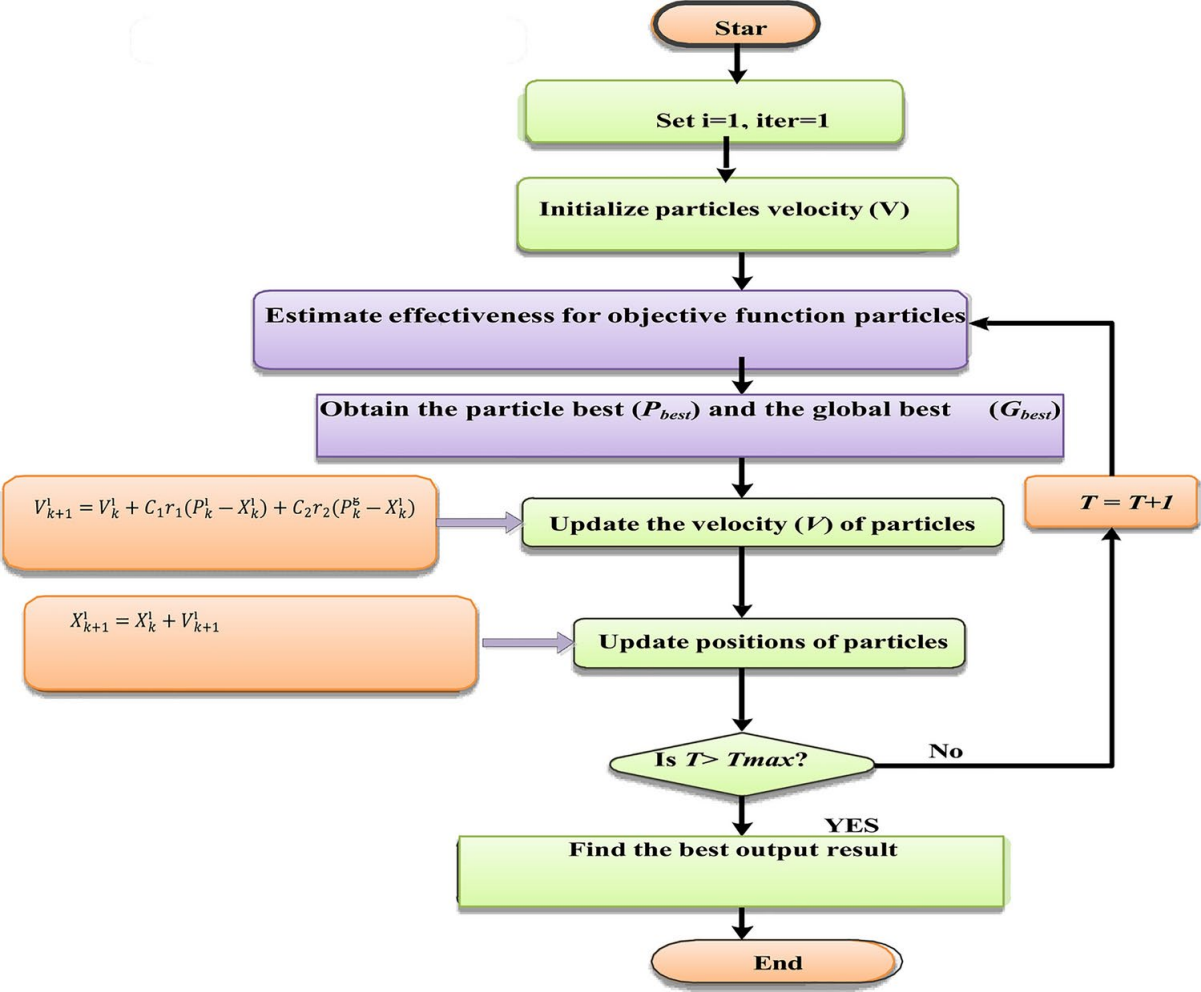
Flowchart of PSO Algorithm.

PSO algorithm flowchart is shown in Fig. 8, which afford the most information about this algorithm and how this algorithm works, and Table 3 shows the parameters of PID controller.

**Table 3:** PSO parameters of PID controller.

PSO parameters	Parameter of voltage regulator			Parameter of current regulator		
	${K}_{P}$1	${K}_{I}$1	${K}_{D}$1	${K}_{P}$2	${K}_{I}$2	${K}_{D}$2
Line to line fault	8.955	2.156e3	0.0050	3.0864	42.2778	4.855e-4

## PID controller based PSO

**Fig. 9:**
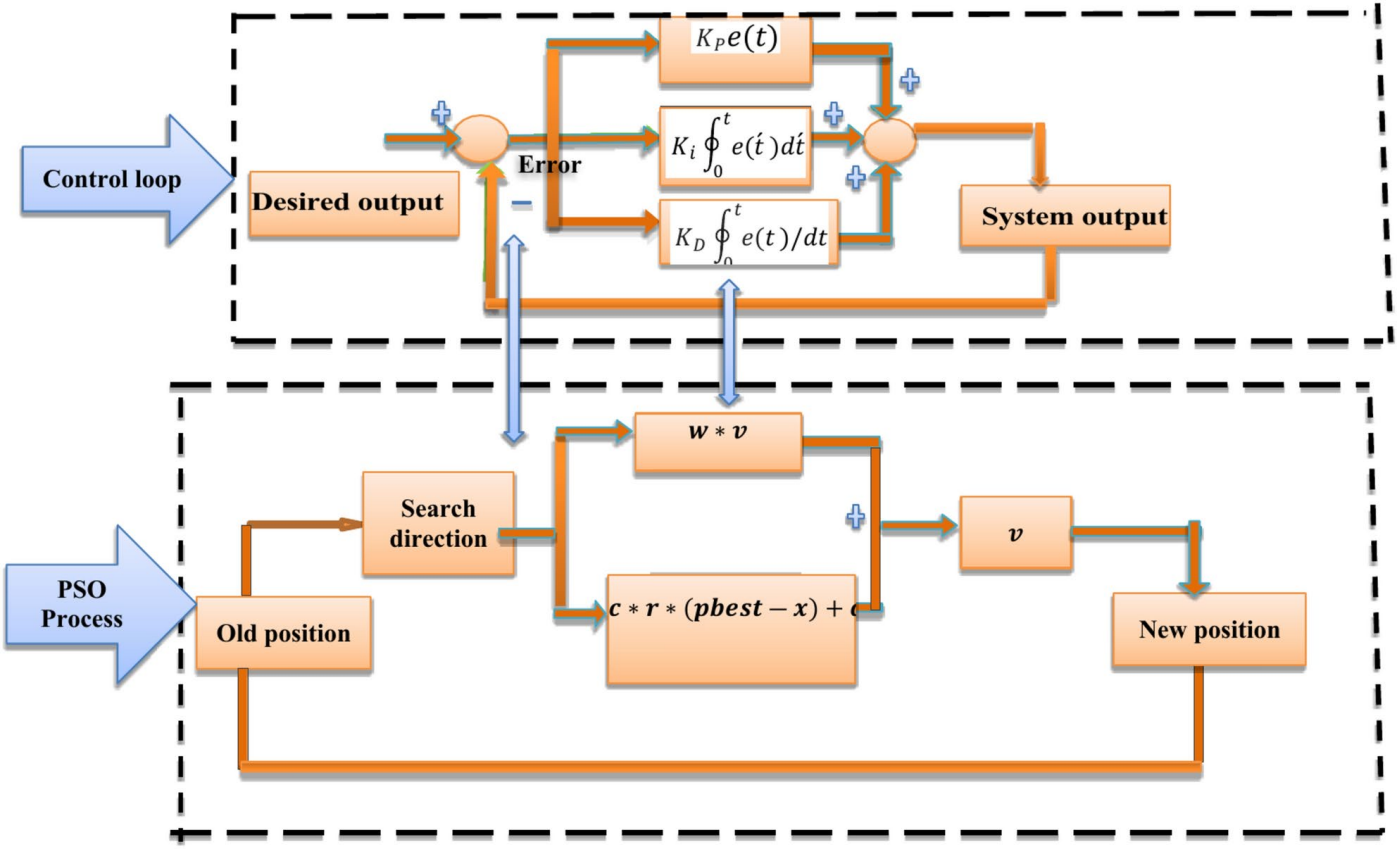
Connection between control loop PID and PSO process.

Figure 9 demonstrates the link between PID controller and the PSO process. The control system aims to calculate the difference between measured value and desired value and then apply an error correction automatically during function of control^60^. In a feedback control system, function of control will try to reduce the error by detecting the control variable u(t). To reach this aim PID controller-based control function applies a correction u(t) based on the current (proportional term), past (integral term), and future(derivative term) of the error e(t), as shown in Eq. (7). In the PSO process, the method of optimization aims to search for the global best solution in the problem space iteratively by updating the functions The velocity characterizes final search direction based the past cumulative momentum and the current search direction, that are obtained by calculating the difference between the best position and the current position of the particle.

In the process of optimization, the positions of particles will be updated continuously. The update stops when the change of the current search direction is small enough that the process of optimization approaches optimality. As shown in Fig. 9, the PSO process (control loop) updates the process (loop) based on the search direction (difference error) between the new value and old value^61^. The final goal of the PSO process is to reduce the search direction $e\left( {\mathop t\limits^{\prime } } \right).$ $\left( {{\text{(error}}\;e\left( {\mathop {{\text{ }}t}\limits^{\prime } } \right)} \right)$. Therefore, it is reasonable to associate the error e(t) in PID control with the search direction $e\left( {\mathop t\limits^{\prime } } \right).$ in PSO. The basic difference is that the PID controller calculates the correction utilization system error $e\left( t \right)$ while PSO calculates its search direction by the distance between the global value and current value.

## STATCOM configuration

**Fig. 10:**
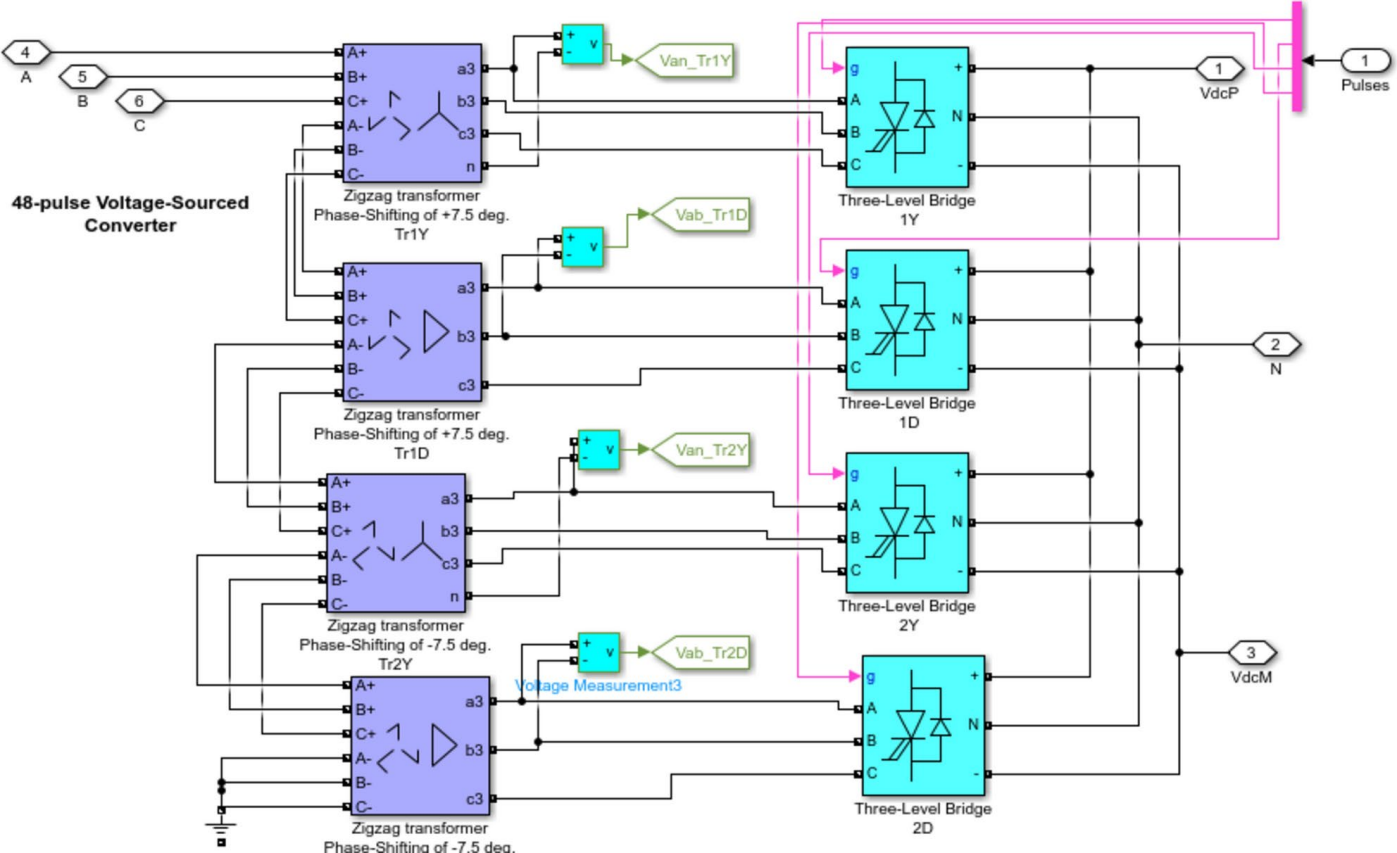
Structure of 100MVAR based on 48 pulse.

Figure 10 clears the structure of the 48-pulse STATCOM circuit, which is used to adjust voltage and improve LVRT capacity. 100 MVAR STATCOM is linked to the grid at the PCC bus. The basic aim of STATCOM is regulating the voltage at the PCC bus by controlling the amount of reactive power absorbed or injected from the grid. It includes three-level GTO inverters connected to four three-phase shifting transformers presenting a phase shift of ± 7.5°. The DC-link of STATCOM was linked with 4 inverters, and the voltage from inverters was provided secondary windings of zigzag phase shifting transformer. The zigzag phase shifting transformer arrangement neutralizes all odd harmonics up to the 45th harmonic. Also, it provides isolation between ground and component and provides allow impedance to zero sequence currents^62,63^.

### Control circuit of STATCOM device

**Fig. 11:**
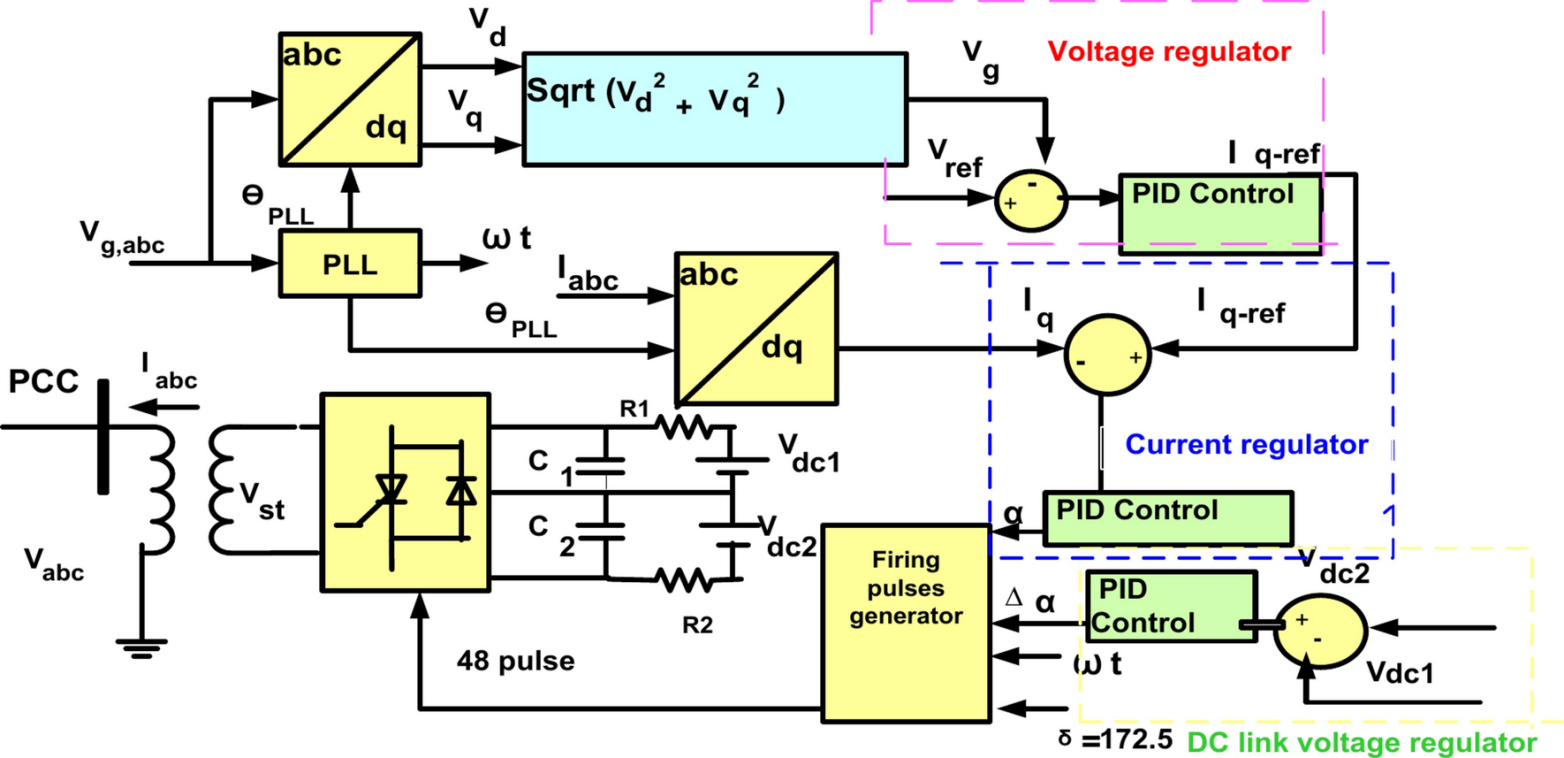
Configuration STATCOM control with PID.

STATCOM is a FACTS device connected in shunt with the network. It is used to compensate for reactive energy^64^. It contains a coupling transformer, DC source, and Voltage Source Inverter (VSI), connected in parallel with the system^65^. Voltage source inverter containing power electronic devices (IGBT or GTO). The voltage of DC input from the capacitor was transformed to group from the controller voltage of three-phase AC output by VSI. The device of STATCOM could work in inductive mode or capacitive mode based distinction between the voltage of PCC and the reference voltage. If the magnitude voltage of PCC reduces, the leading current is injected STATCOM to the electrical network at PCC, but a lagging current in the inductive mode is injected STATCOM to the electrical network at PCC^65,67,68^. Essential component of V_st_ was proportional to V_dc_. Operation of VSI control begins with system measurement that gets dc voltage of capacitor V_dc_, The reactive current I_abc_ of STATCOM, and voltage of bus line V_gabc_, are shown in Fig. 11 @@^66–69^_._ Also in Fig. 11, Phase Locked Loop (PLL) produces coinciding signal (θPLL). Loop of voltage regulating, actual voltage called V_g_ at PCC was contrasted to reference value of voltage V_ref_ and distinction was applied to PID control to produce I_qref_ called reference reactive current. In loop of regulation the current contrasted absorbing or injecting reactive current I_q_ with I_qref_ that called reference value to generate desirable phase angle $\left(\alpha \right)$. Conduction angle (δ) is fixed at 172.5 degree to decrease 23rd and 25th harmonics of produced voltage. DC capacitor voltage positive and negative is preserved equal utilizing offset (Δα) from DC voltage regulating as shown in following equations^69^.

10 $$P_{g} = 3\left( {\frac{{v_{g} v_{st} }}{{x_{tr} }}} \right){\text{sin}}\left( \alpha \right)$$

11 $$Q_{g} = 3\left( {\frac{{v_{g} v_{st} }}{{x_{tr} }}} \right)\cos \left( \alpha \right) - \frac{{v_{g}^{2} }}{{x_{tr} }}$$

12 $$I_{qref} = \left( {V_{ref} - V_{g} } \right)(K_{P} + \frac{{K_{I} }}{{\text{S}}} + K_{D} {\text{s}})$$

13 $${\upalpha } = \left( {I_{qref} + I_{q} } \right)(K_{P} + \frac{{K_{I} }}{{\text{S}}} + K_{D} {\text{s}})$$

14 $${\Delta }\alpha = \left( {V_{dc2} - V_{dc1} (K_{P} + \frac{{K_{I} }}{{\text{S}}} + K_{D} {\text{s}}} \right)$$

where ${v}_{g, }{ v}_{st}$: Grid voltage and STATCOM voltage respectively; ${x}_{tr}$: Leakage reactance of coupling transformer between VSI and the grid; $\alpha$ : Phase angle between STATCOM voltage and grid voltage; ${I}_{qref,}{I}_{q}$: Reference reactive current and reactive current; ${K}_{I}$,${K}_{D},$ ${K}_{p}$: Parameters of PID controller.

**Fig. 12:**
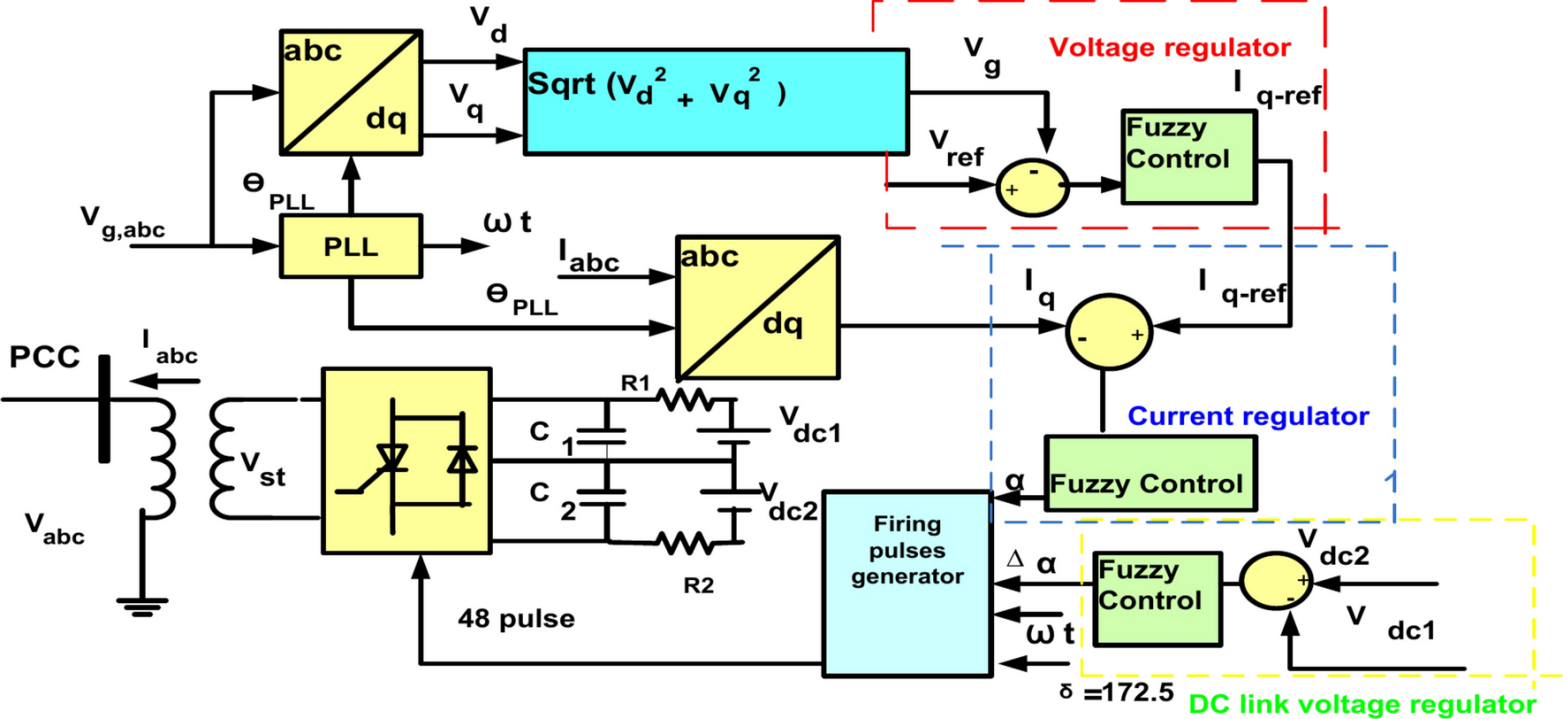
Configuration of STATCOM control with fuzzy logic controller.

Also, PSO is used to get the best parameter of PID controller. In Fig. 12 FLC is applied to STATCOM to improve performance of result.

## Simulation study and results

Figure 1 proposes a Simulink model of PV/wind hybrid model linked with network. Hybrid system produces 10 MW of active power divided into 9 MW from the wind farm and 1 MW from the PV station. STATCOM connected at PCC bus to network for compensating reactive power to hybrid system and the load that connected in the grid during faults grid occur. The load energizes with 9.2 MW. Line-to-line asymmetrical fault is applied to the grid. Compression between STATCOM PID control and FLC is studied for improving performance of STATCOM as shown in the results and achieve voltage stability.

### Case study line to line fault (LLF)

The Line to Line Fault (LLF) is stratified at 0.5 s and removed at 0.7 s in grid fault effect on wind bus 575 V, bus 25 kV, and also effect on PV station. Voltage sagging occurs on the voltage of the wind bus and PV bus and also impacts on DC Link voltage of wind and PV so when STATCOM is linked for compensating voltage, active and reactive energy of wind, PV and decrease oscillations of DC link voltage. Also, comparisons between different types of control like PID control and FLC are studied to enhance LVRT.

#### DFIG model

**Fig. 13:**
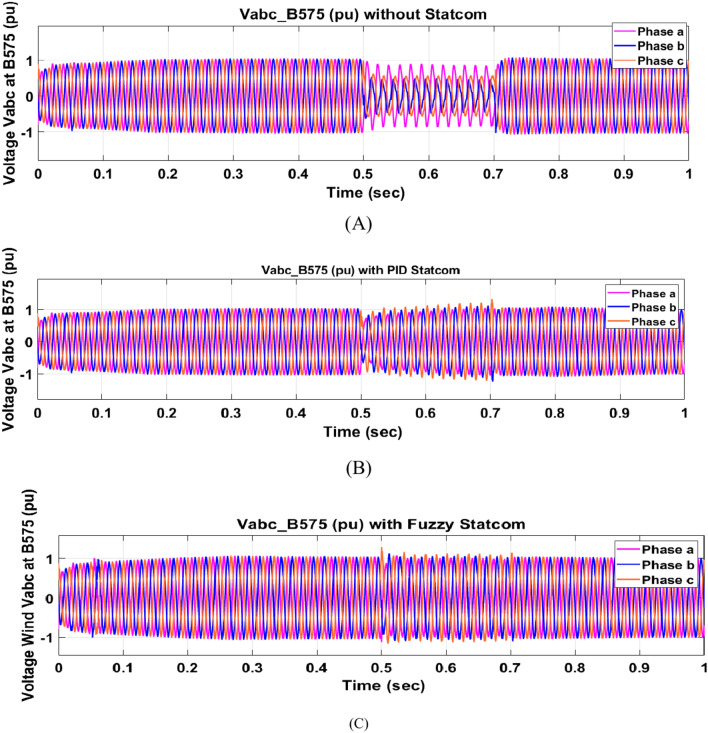
(**A**) The voltage at Bus 575 through LL fault without links STATCOM. (**B**) The voltage at Bus 575 with controller PID STATCOM. (**C**) The voltage at B 575 with fuzzy logic control STATCOM.

**Fig. 14:**
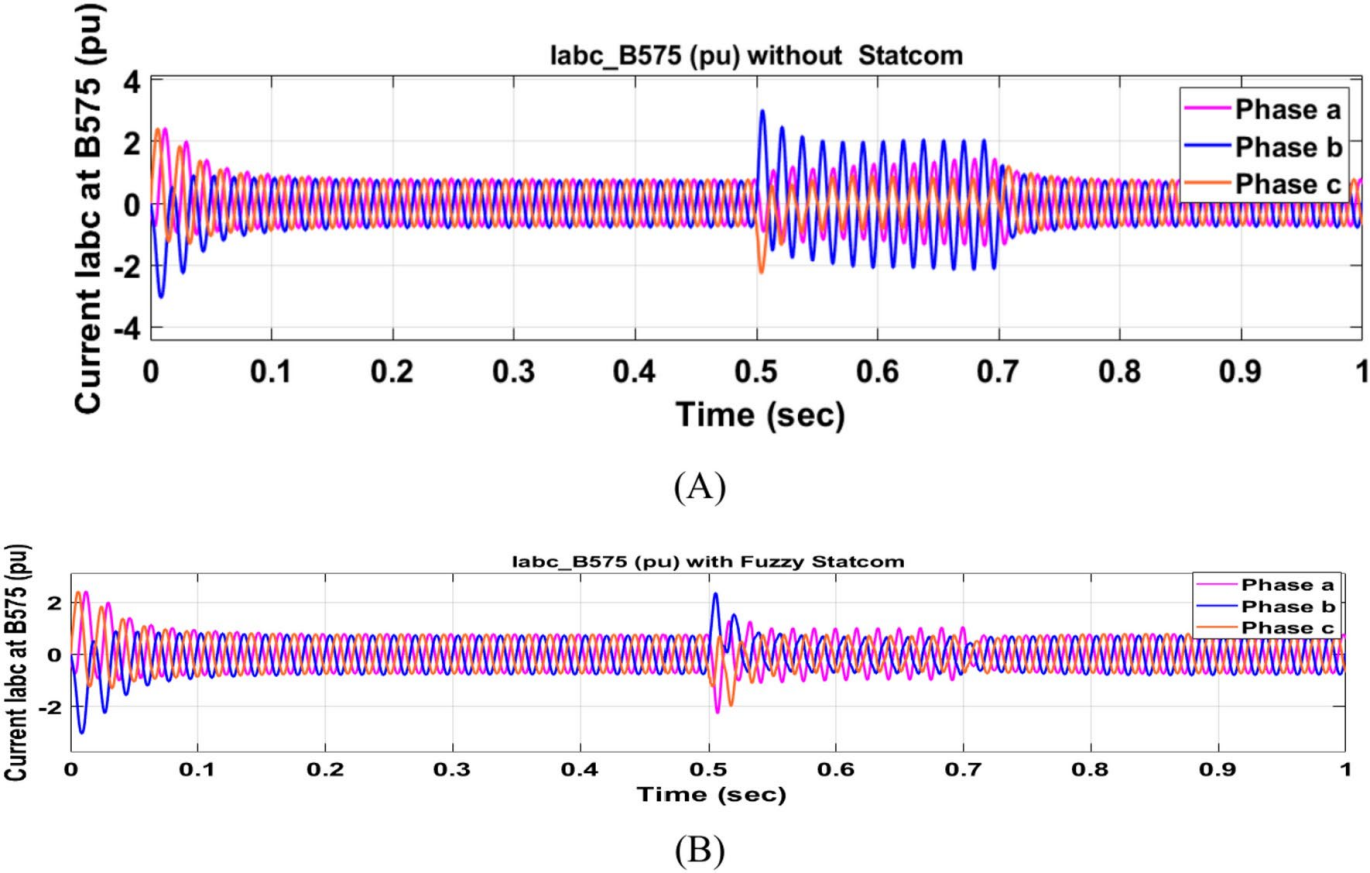
(**A**) The current at Bus 575 through LL fault without links STATCOM. (**B**) The current at B 575 with PID STATCOM controller and current at B 575 with fuzzy logic control STATCOM.

**Fig. 15:**
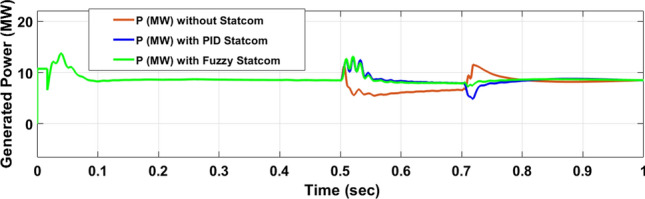
Active power produced at Bus 575 during LL fault without links STATCOM, with PID STATCOM control and with FLC STATCOM.

**Fig. 16:**
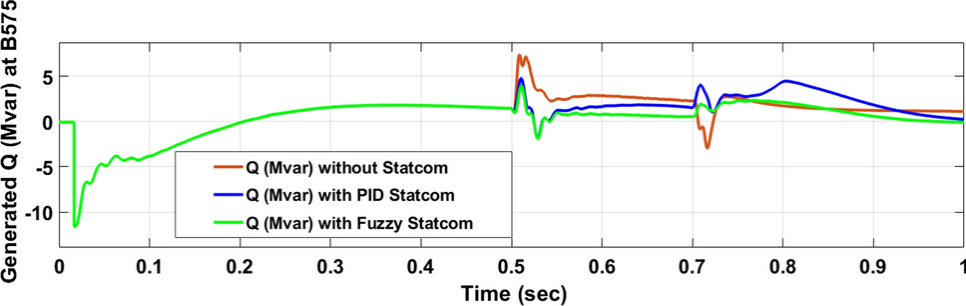
The reactive power produced at Bus 575 through LL fault without links STATCOM, with PID STATCOM control and with FLC STATCOM.

**Fig. 17:**
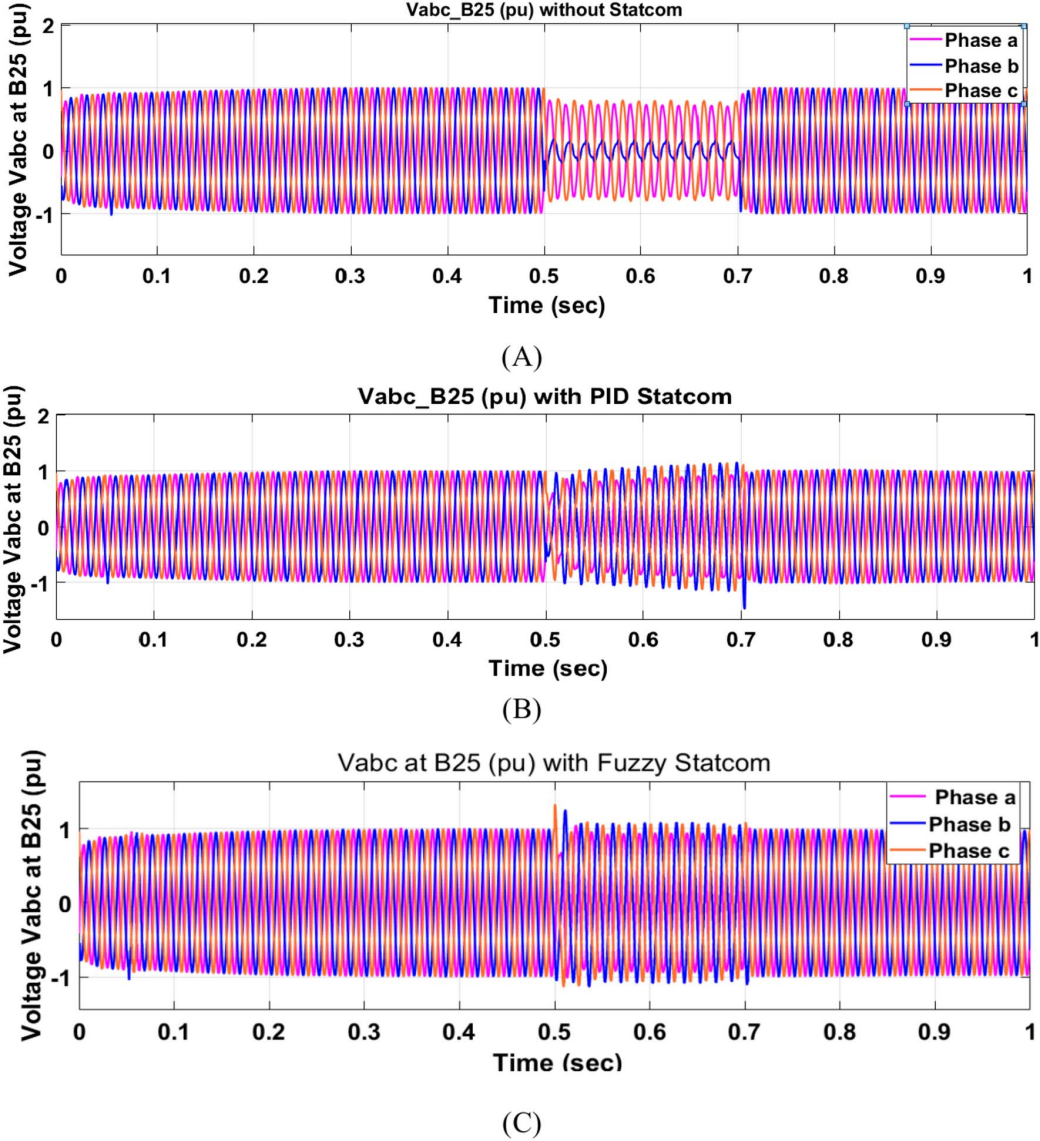
(**A**) The voltage at Bus 25 through LL fault without links STATCOM. (**B**) The voltage at Bus 25 with PID STATCOM control. (**C**) The voltage at Bus 25 with FLC STATCOM.

**Fig. 18:**
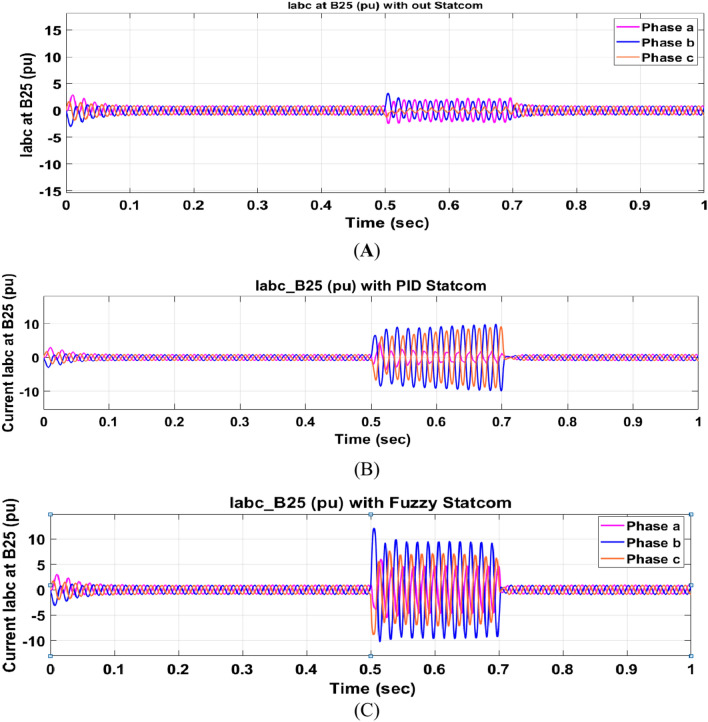
(**A**) The current at Bus 25 through LL fault without links STATCOM. (**B**) The current at B 25 with PID STATCOM control. (**C**) The current at B 25 with FLC STATCOM.

**Fig. 19:**
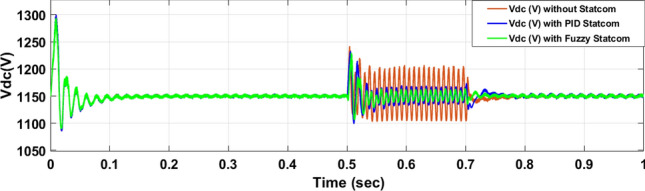
The voltage of DC link at Bus 575 through LL fault without links STATCOM, with PID STATCOM control and with FLC STATCOM.

**Fig. 20:**
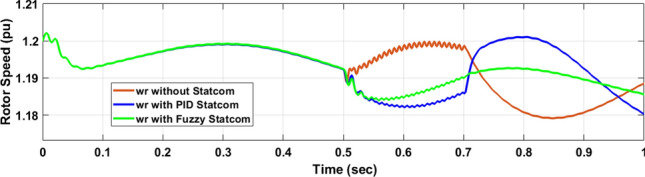
Rotor speed of DFIG through LL fault without links STATCOM, with PID STATCOM control and with FLC STATCOM.

Voltage sagging occurs at bus 575 V during the period of fault LL between 0.5 and 0.7 s as shown in Fig. 13A when not linked to STATCOM and when PID, Fuzzy STATCOM connected voltage sagging was mitigated as shown in Fig. 13B,C. Also Fig. 14A,B shows current before and after linked STATCOM to mitigate voltage swell. Figure 15 shows active power before and after linking STATCOM with PID, Fuzzy. Figure 16 shows reactive power before and after linking STATCOM with PID, Fuzzy. Voltage sag occurs at bus 25 kV when STATCOM is not linked as shown in Fig. 17A and is mitigated when STATCOM is linked as shown in Fig. 17B, C. Figure 18A–C shows the current before and after connecting STATCOM Fig. 19 shows the DC link voltage before and after linking STATCOM with PID, Fuzzy. Also, Fig. 20 shows the rotor speed of DFIG during LL before and after STATCOM. Compression in Table 4 shows that.

**Table 4:** Comparisons of the results through LLF at B 575 V.

Comparisons state	The active power (MW) minimum–maximum	The reactive power (MVAR) minimum–maximum	Voltage of DC link
Without STATCOM	5.5–11.5	− 2.85 to 7.4	1208
With PID STATCOM	5.3–12.44	− 1.88 to 4.7	1173
With Fuzzy STATCOM	8–13	− 1.88 to 3.5	1155

#### PV model

**Fig. 21:**
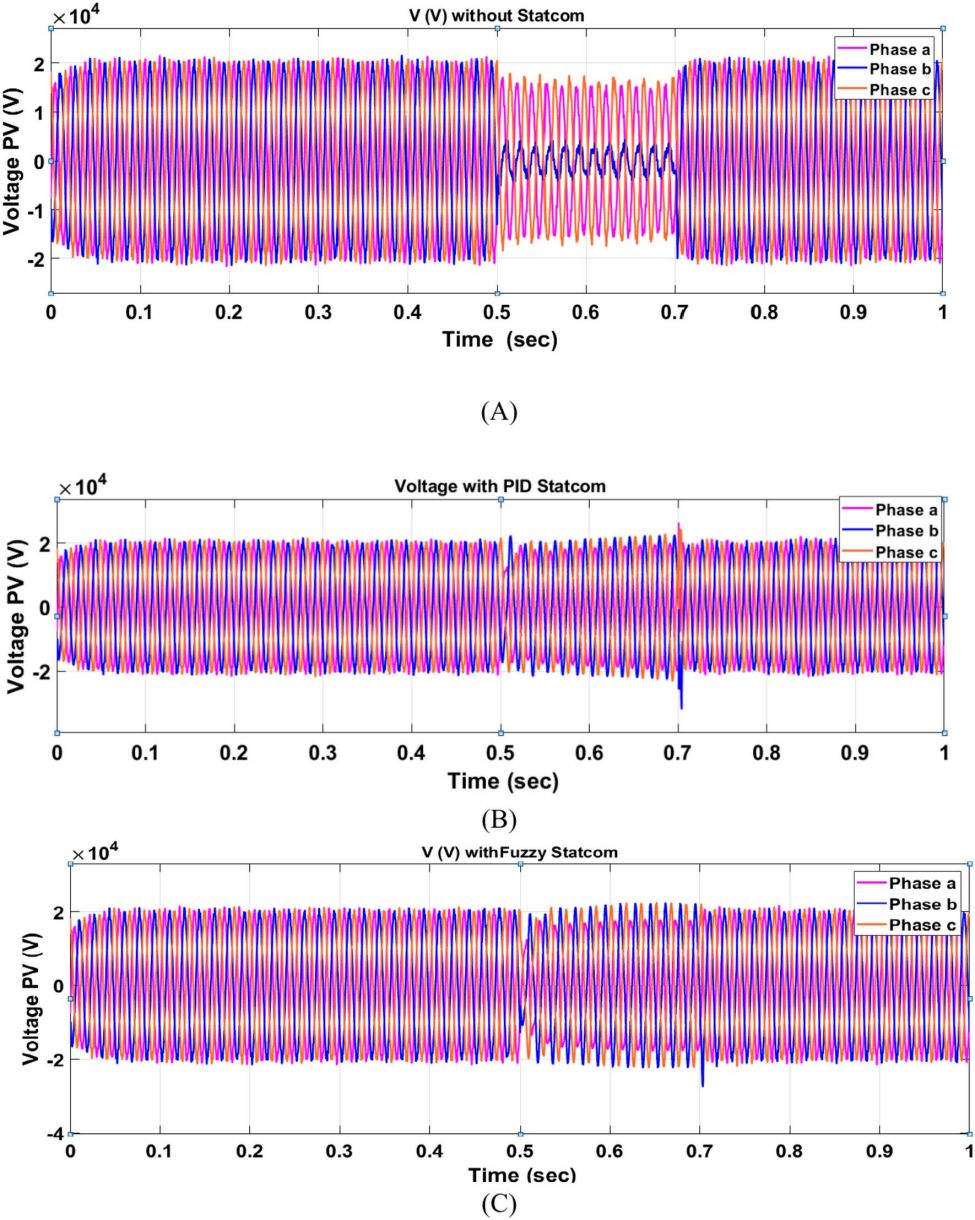
(**A**) PV voltage through LL fault without STATCOM. (**B**) PV voltage with PID STATCOM control. (**C**) PV voltage with FLC STATCOM.

**Fig. 22:**
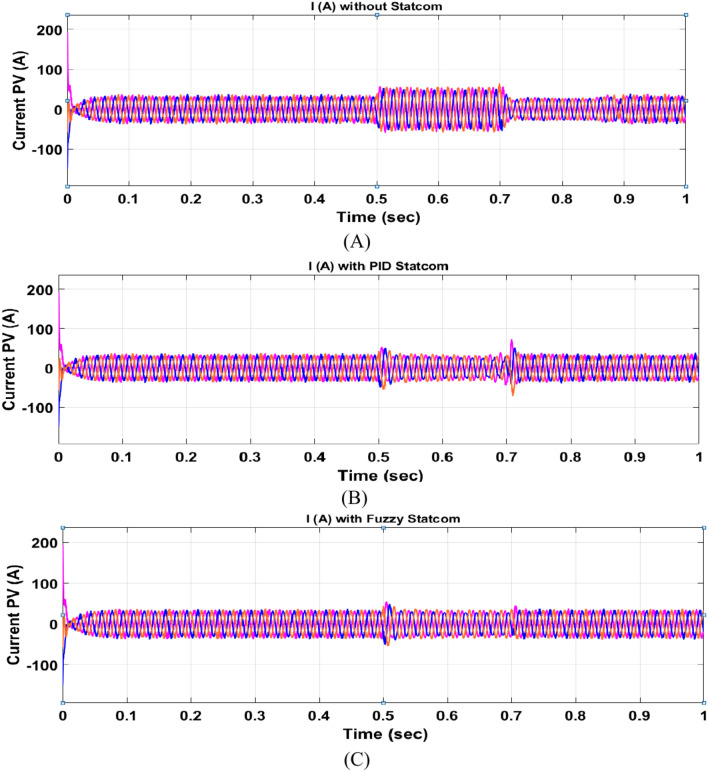
(**A**) PV current through LL fault without STATCOM. (**B**) PV current with PID STATCOM control. (**C**) PV current with FLC STATCOM.

**Fig. 23:**
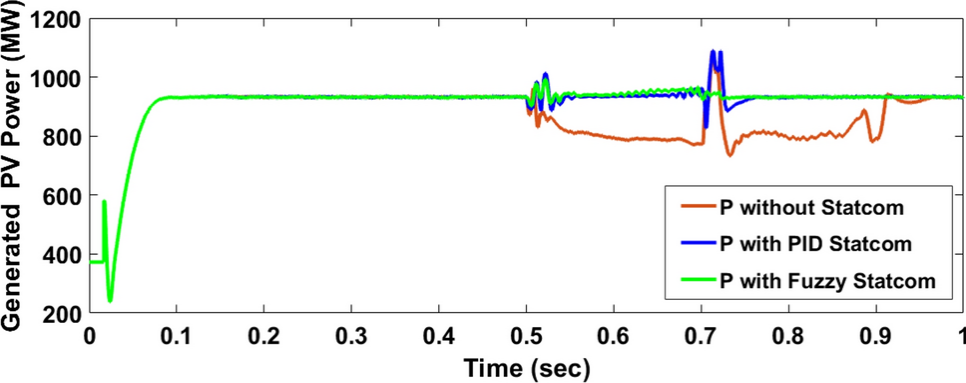
PV active power generated through LL fault without links STATCOM, with PID STATCOM control and with FLC STATCOM.

**Fig. 24:**
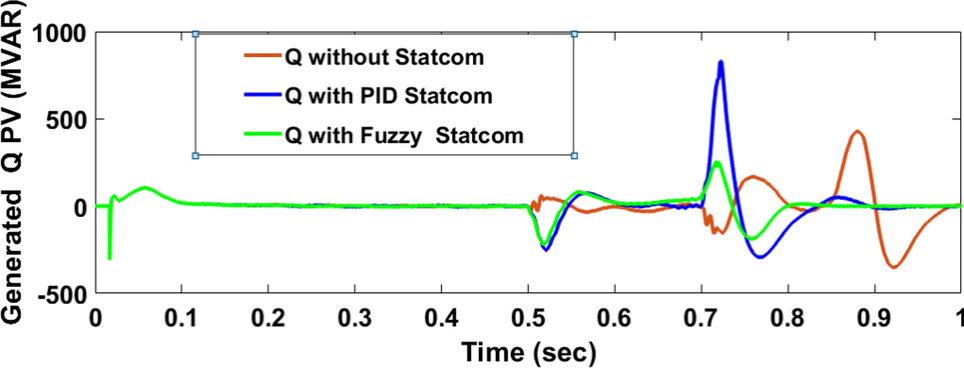
PV reactive power generated through LL fault without links STATCOM, with PID STATCOM control and with FLC STATCOM.

**Fig. 25:**
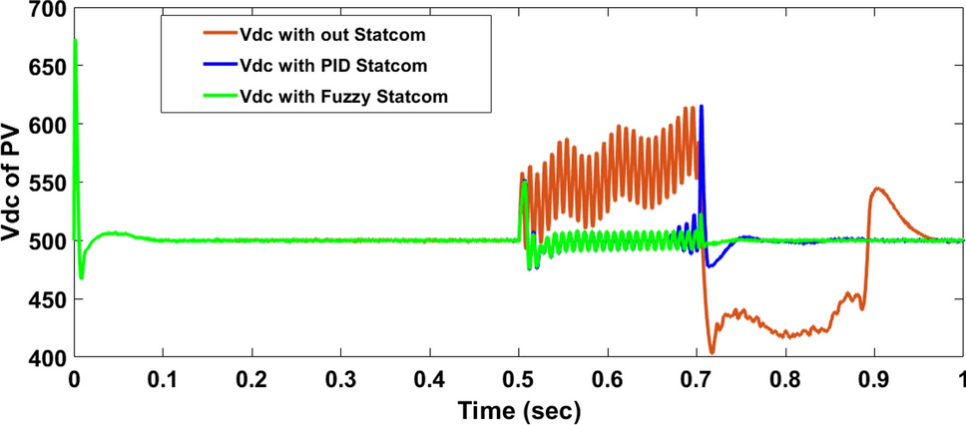
The Voltage of DC link Voltage of through LL fault without links STATCOM, with PID STATCOM control and with FLC STATCOM.

Voltage sagging occurs at PV bus at the period of fault as shown in Fig. 21A and when PID, and Fuzzy STATCOM linked voltage sag was mitigated as shown in Fig. 21B,C. Also Fig. 22A–C shows current before and after linked STATCOM to mitigate voltage swell. Figure 23 shows active power before and after linked STATCOM with PID and Fuzzy. Figure 24 shows reactive power before and after linked STATCOM with PID, Fuzzy. Figure 25 shows DC link voltage before and after connected STATCOM with PID and Fuzzy as shown in compression in Table 5.

**Table 5:** Comparisons of the results through LLF at PV Bus.

Comparisons state	The active power (KW) minimum–maximum	The reactive power (KVAR)	Voltage of DC link minimum–maximum
Without STATCOM	771–1056	− 155 to 169.6	406–613
With PID STATCOM	838–1070	− 254.5 to 824	477–614
With Fuzzy STATCOM	922–995	− 210 to 214	479–551

#### Hybrid PV/wind

**Fig. 26:**
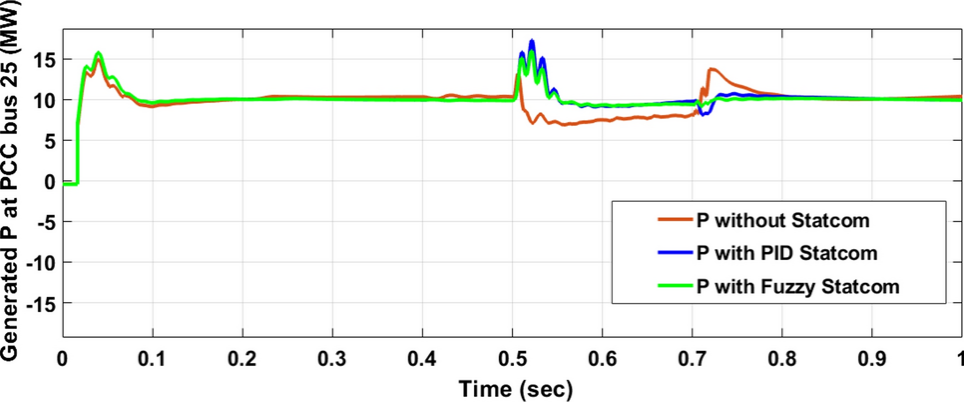
Generated active power at Bus PCC through LL fault without links STATCOM, with PID STATCOM control and with FLC STATCOM.

**Fig. 27:**
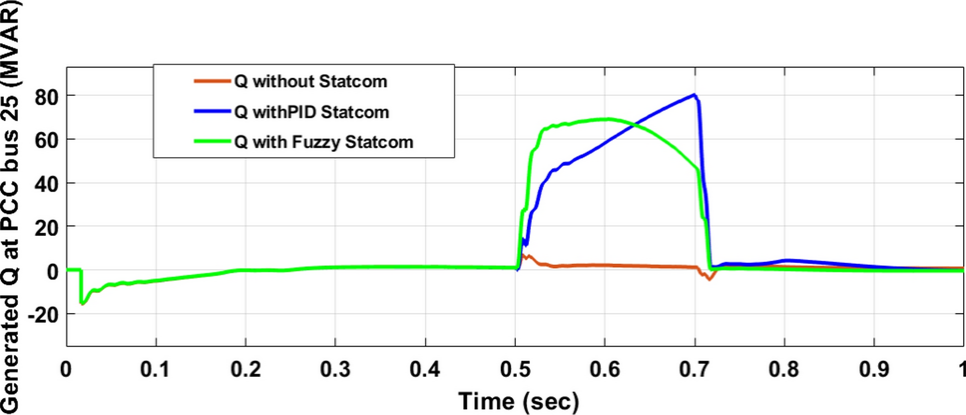
Generated reactive power at Bus PCC through LL without links STATCOM, with PID STATCOM control and with FLC STATCOM.

**Fig. 28:**
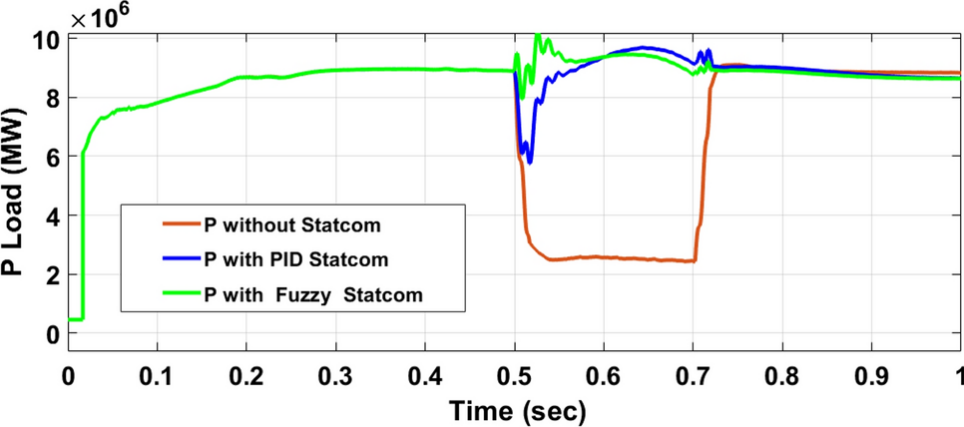
Load active power through LL fault without links STATCOM, with PID STATCOM control and with FLC STATCOM.

**Fig. 29:**
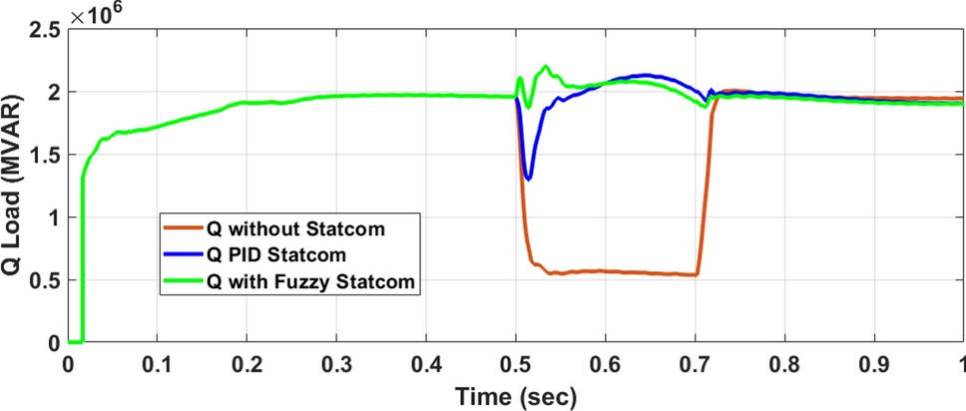
Load Reactive power through LL fault without links STATCOM, with PID STATCOM control and with FLC STATCOM.

**Fig. 30:**
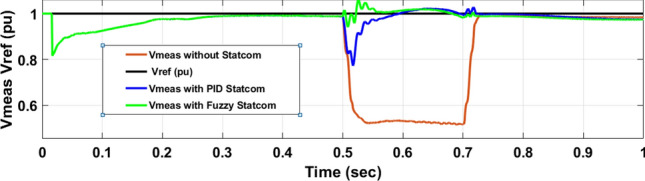
V_meas_, V_ref_ of STATCOM through LL fault without links STATCOM, Vref, with control PID STATCOM and with FLC STATCOM.

**Fig. 31:**
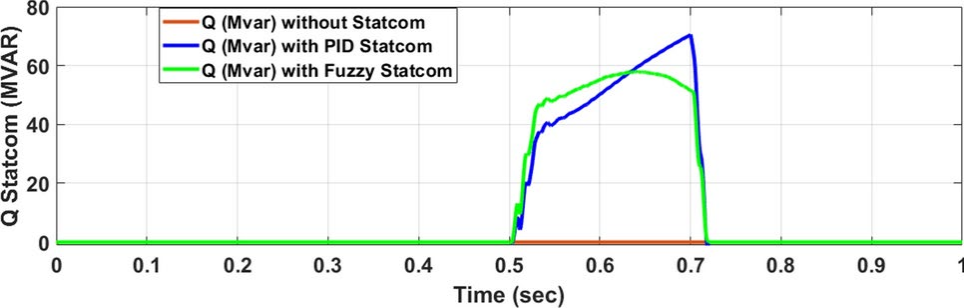
Generated reactive power at STATCOM through LL fault without STATCOM, with control PID STATCOM and with FLC STATCOM.

Figure 26 shows the Active power of hybrid PV/wind produced at B 25 before and after linked STATCOM in case PID and Fuzzy. Figure 27 shows reactive power before and after linked STATCOM with PID and Fuzzy. Figure 28 shows active power of load before and after linked STATCOM. Figure 29 shows reactive power of load before and after linked STATCOM with PID and Fuzzy. Figure 30 shows V_meas_ V_ref_ where V_meas_ is voltage measured and V_ref_ is voltage reference at bus STATCOM before and after STATCOM linked with PID and Fuzzy. Also Fig. 31 shows reactive power produced at bus STATCOM with PID and Fuzzy. Table 6 offers comparisons among PID and FLC to improve LVRT. Also, Table 7 offers comparisons among PID controller and FLC at STATCOM Bus to enhance LVRT.

**Table 6:** Comparisons of results through LLF at Hybrid PV/wind B 25 kV.

Comparisons state	The active power (MW) minimum–maximum	The reactive power (MVAR)	P load (MW)	Q (MVAR)
Without STATCOM	7.4–13.2	0	2.5	0.56
With PID STATCOM	8.58–17.1	62	4.2–10	1–2.2
With fuzzy STATCOM	9.5–15.8	69	8.3–10	1.93–2.2

**Table 7:** Comparisons of results through LLF at STATCOM Bus.

Comparisons state	Vmeas	Vref	Reactive power
Without STATCOM	0.53	1	0
With PID STATCOM	0.69–1.05	1	54.5
With Fuzzy STATCOM	0.96–1.05	1	62.6

## The limitations and future work

1. In the case of change in the load of the model, the parameter of the controller will change.
2. In the case change parameter of the three-phase transmission line with the PI section, the parameter of controller change.
3. In the case change in the value of the active power of the wind turbine and the power of the PV station, the parameter of the controller change.
4. In the case change the capacitance value of STATCOM, the parameter of the controller will change.

## Conclusion

This paper studies the LVRT Issue that occurs due to line-to-line fault and its impact on a hybrid system consisting of a 9 MW wind farm and 1 MW PV station. So, the STATCOM device is used to solve this problem. The proposed STATCOM is used to compensate for reactive power and mitigate voltage dip. The PSO optimization method is applied to the PID controller to get the best PID parameter. The simulation results shows the effectiveness of suggested STATCOM in mitigation voltage dip, compensating reactive and active power of PV and wind farm, and protecting DC- Link voltage from overvoltage. In the case of line-to-line fault, STATCOM with FLC compensates for voltage by 96% compared with PID which compensates for voltage by 69%. STATCOM with FLC gives 62.6MVAR reactive power compared with STATCOM with PID, which gives 54.5 MVAR reactive power. Comparison between STATCOM PID controller and Fuzzy logic control is studied to achieve the best performance for STATCOM but FLC gives better performance than PID controller in enhancing LVRT capability, and power quality.

## Data Availability

All data generated or analyzed during this study are included in this published article.
